# The process of behavioral change in individuals who are uninterested in health: a qualitative study based on professional health knowledge

**DOI:** 10.1265/ehpm.22-00072

**Published:** 2022-07-28

**Authors:** Takashi Shimazaki, Hiroto Okoshi, Takashi Yamauchi, Koji Takenaka, Machi Suka

**Affiliations:** 1 Department of Public Health and Environmental Medicine, The Jikei University School of Medicine, 3-25-8 Nishi-Shimbashi, Minato-ku, Tokyo 105-8461, Japan; 2 Faculty of Human Sciences, Waseda University, 2-579-15 Mikajima, Tokorozawa-shi, Saitama 359-1192, Japan

**Keywords:** Uninterested, Pre-contemplation, Qualitative, Health behavior, Behavior change, Gateway, Readiness, Transtheoretical model

## Abstract

**Background:**

In Japan and elsewhere, there is major concern over individuals who are uninterested in health and reluctant to change their health behaviors. While previous studies have investigated cognitive and behavioral characteristics in this population, there is limited evidence on whether they recognize the significance of health, nor is it clear how to motivate necessary behavior changes. This study identified specific characteristics of positive psychological and behavioral change in individuals who were uninterested in health, then constructed a model for their behavior change process, as advised via professional health expertise in the Japanese context.

**Methods:**

This qualitative survey study was conducted among 86 health professionals (public health nurses, registered dieticians, and city/prefectural employees). These participants reported their demographic characteristics (gender, age, job, and length of service) and entered free descriptions concerning perceived cognitive and behavior changes in individuals who were uninterested in health. Finally, we thematically analyzed the contents on psychological/behavioral change and constructed a thematic map.

**Results:**

We obtained 409 relative descriptive codes and four main themes, including (1) Health awareness: Recognize the significance of health via personal experience and/or illness among family/friends; (2) Psychological readiness: Preparative psychological state toward health behavior; (3) Gateway behavior: Precursory behavior leading to health behavior; and (4) Health behavior: Traditional healthy lifestyle behavior, with 45 subthemes. We constructed the abovementioned thematic map according to the Transtheoretical Model. Herein, health awareness may catalyze changes in health behavior, while changes in both psychological readiness (e.g., new interest in health behaviors and attitude toward appearance) and gateway behaviors (e.g., new points of discussion and information gathering) may arise before changes in health behavior.

**Conclusions:**

This study clarified positive cognitive and behavior changes in individuals who were uninterested in health and elucidated their behavior change process. As behavior changes in such individuals tend to be rigid, they are often left behind by health care systems and programs. In this regard, we identified pertinent cognitive and behavioral characteristics during the behavior change process and constructed a relevant model. These findings should be useful in developing interventions that can motivate the desire for behavior change.

**Supplementary information:**

The online version contains supplementary material available at https://doi.org/10.1265/ehpm.22-00072.

## Graphical Abstract

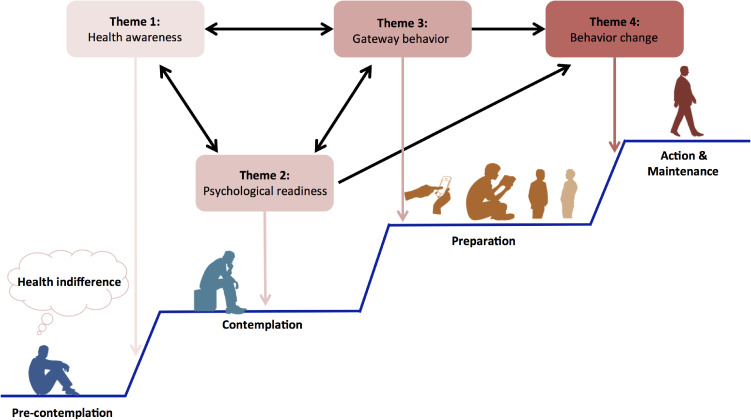

## Introduction

The need to prevent non-communicable diseases and increase the quality of life (QoL) through positive health behavior is a significant health issue around the globe [1]. Specifically, health behaviors such as physical activity [2], healthy eating [3], controlled alcohol consumption [4], stress management or mental health promotion [5], and smoking cessation [6] contribute to physical and psychological health while preventing disease [7]. However, there are major concerns over individuals who are indifferent toward health and do not intend to engage in healthy lifestyle behaviors, otherwise known as uninterested people toward health (UPH).

In the Japanese context, the Ministry of Health, Labour and Welfare [MHLW] [8] reported that 10.5% of females and 16.5% of males do not want to improve their unhealthy eating behaviors. Similarly, 11.1% of females and 13.9% of males had no interest in physical activity, nor did they intend to engage in such practices. In the Netherlands, Ronda, Van Assema, and Brug [9] reported that 29.6% of participants were inactive and had no intention to change their lifestyles. In Korea, a report showed that 72% of smokers did not intend to quit [10]. A study among students in Inner Mongolia investigated multiple negative health behaviors, including physical inactivity, unhealthy eating, poor stress management, alcohol consumption, and smoking, thus finding that 4–14% of respondents reported unhealthy lifestyles and did not intend to change [11]. In Japan and many other countries, there is a clear and urgent need for policies and practices that encourage UPH to engage in healthy lifestyle behaviors.

Several previous studies have focused on identifying the underlying reasons why UPH do not practice health behaviors, including their relevant behavioral characteristics. According to a comprehensive research review, the UPH population is characterized by the following: does not practice health-related behavior, no intention to change unhealthy lifestyle, and uninterested in health status or information, including discussions with friends or family members, despite their health condition [12]. Under the Transtheoretical Model (TTM) [13], UPH are regarded as persons in the pre-contemplation stage. Prochaska and DiClemente [13] defined the pre-contemplation stage as that in which individuals are unaware, unwilling, or discouraged when changing a particular problem behavior. The characteristics of the pre-contemplation population include the lack of self-efficacy toward health behavior change [14] and an unbalanced perception of the benefits (i.e., pros) and psychological burdens (i.e., cons) associated with engagement in health behavior [15]. Under another framework, self-determination theory (SDT) [16] entails that UPH are in the motivational state of “amotivation,” in which they either do not engage or have no motivation to engage in health behavior. Ryan et al. [17] argued that populations with amotivation lack autonomy, competence, and relatedness. Moreover, these populations have four-dimensional irrational beliefs about health promotion, including a suspicion of health promotion programs, the lack of confidence in their efforts, a sense of insufficient capacity, and helplessness [18]. Other relative SDT research has shown that populations with amotivation have higher depressive symptoms, anxiety, negative affective states, lower QoL and vitality, and inactivity [19]. Surprisingly, a national survey in Japan found that the most common answer and primary cause for health indifference was “no reason” [8].

Extensive efforts have been dedicated to documenting the assumed rate of UPH and its characteristics. By contrast, relatively little is known about whether UPH recognize the importance of health and the need to change their unhealthy lifestyles (i.e., positive psychological and behavioral change processes). This knowledge may be useful in developing intervention strategies for UPH. As such, this study aimed to identify specific substances of positive psychological and behavioral changes and construct a model for the cognitive and behavior change process in Japanese UPH, as advised by qualitative evidence and empirical knowledge held by health professionals who support affected persons on a daily basis.

## Methods

### Qualitative approach and research paradigm

This study followed the Standards for Reporting Qualitative Research (SRQR) guidelines [20]. Additional file 1 contains the SRQR checklist. As mentioned, we constructed a model for the behavior change process in UPH, which necessitated a phenomenological qualitative approach. We also conducted a literature review, data analysis, and interpreted the results (the authors are Ph.D. level researchers and medical doctors specializing in health psychology, epidemiology, and public health).

### Context and setting

We recruited health care and promotion professionals from the community setting. This was done because professional health practitioners in Japan are legally obliged to encourage UPH according to the Health Promotion Act [21]. In this context, they communicate with and provide health guidance to UPH on a daily basis. These health professionals should thus have a good understanding of UPH cognitive and behavioral characteristics.

The qualitative survey was conducted in Kanagawa prefecture, an area adjacent to Tokyo, the capital city of Japan, and Tochigi prefecture, Tokyo’s northern suburb area. We recruited participants via snowball sampling. The researchers consulted with three familiar health professionals in management positions, who were informed about the research aims and privacy policy. They then shared this information with their acquaintances and coworkers. Three primary collaborators independently recruited participants near their working area (Collaborator A: Yokohama city, the eastern metropolitan area of Kanagawa prefecture; Collaborator B: The wide western suburban area of Kanagawa prefecture; Collaborator C: Utsunomiya city in Tochigi prefecture).

A required minimum sample size was assumed based on the methodological guidelines for thematic analysis [22]. Based on the lowest prevalence of interest theme among study participants = 20% and appearance of relevant description = 75%, the adjusted prevalence was supposed at 15%. According to previous research [23], the desired number of theme instances was assumed as four (i.e., emotional, psychological, behavioral, and background factors). The minimum sample size at 80% power was *n* = 44.

Regarding the circumstances of recruitment, 109 participants (Yokohama city, the eastern area of Kanagawa prefecture: n = 7; western area of Kanagawa prefecture: n = 67; Tochigi prefecture: n = 35) were recruited and responded to the questionnaire survey. The inclusion criteria were as follows: (1) hold a full-time public health-related job, (2) answered their professional job, and (3) described relative cognitive or behavioral description in at least one sentence. Meanwhile, 23 participants were excluded for the following: part-time instructors who were university student staff (*n* = 12), did not answer their professional job (*n* = 5), and did not respond to the qualitative question (*n* = 6). Thus, data from a total of 86 participants (Yokohama city, the eastern area of Kanagawa prefecture: n = 6; western area of Kanagawa prefecture: n = 48; Tochigi prefecture: n = 32) were subjected to analysis. Table 1 shows their demographic characteristics. The results of the present study reflected the opinions of female public health nurses and early career (0–9 years) professionals.

**Table 1:** Participant characteristics

**Variables**	***n***	**(%)**	***χ* ^2^**	***df***	***w***
Gender					
Female	79	(91.9)	60.28**	1	0.84
Male	7	(8.1)			
Age (years)					
20–29	16	(18.6)	3.02	3	0.19
30–39	20	(23.3)			
40–49	27	(31.4)			
50–59	23	(26.7)			
Job					
Public health nurse	56	(65.1)	230.84**	7	1.64
Dental hygienist	1	(1.2)			
City or prefectural employee	9	(10.5)			
Registered dietitian	13	(15.1)			
Fitness instructor	4	(4.7)			
Nurse	1	(1.2)			
Teacher	1	(1.2)			
Medical Doctor	1	(1.2)			
Professional work experience (years)					
0–4	21	(24.4)	20.44**	6	0.50
5–9	18	(20.9)			
10–14	9	(10.5)			
15–19	13	(15.1)			
20–24	12	(14.0)			
25–29	4	(4.7)			
30+	5	(5.8)			
No response	4	(4.7)			

**p < .01

*Note*. Effect size indicated bias as small (>0.10), medium (>0.30), and large (>0.50).

### Data collection method and instrument

The survey was conducted from December 2019 to February 2020. Participants answered items on their demographic characteristics (gender, age, job, and length of service). Regarding behavioral and psychological changes in UPH, they were asked the following: “When did you feel there was a positive change in behavior or conversation among UPH?” Immediately before the answer box, they were shown a few examples, such as taking a walk around the neighborhood, being interested in sportswear, expressing concern about calories in a convenience store and restaurant, scrutinizing the health section of a magazine, going outside at least once each day, and buying dental floss. Participants were allowed to give free descriptions. The first author entered the obtained data into the computer in the form of a Word document.

### Data analysis

The risk of bias stemming from participant characteristics was tested via a chi-square goodness-of-fit test by *R* version 3.2.3. This study used the thematic analysis method [24] to identify cognitive and behavioral characteristics. The thematic analysis is useful for exploring unknown components of concepts or factor structures that are matched with a given research subject. Our thematic analysis defined the description classifications as follows: code: short sentences retrieved from descriptive data referring to specific cognitive or behavioral phenomenon, category: integrated data of the codes via similar meaning, subtheme: cognitive or behavioral unit consolidated category, and theme: primary component of the theoretical model synthesized from subtheme. The thematic analysis consisted of the following six-step data analysis process: step 1: reviewing and familiarizing the overall collected law descriptive data, step 2: coding and classifying relevant code (i.e., gateway behavior and cognition), step 3: exploring the central and subthemes of the overall code, step 4: reviewing and confirming the identified theme and generating a thematic map that illustrates the hypothesized relationships among themes, step 5: confirming and defining the theme name and finalizing the thematic map, and step 6: describing the scientific research report [24]. In step 4, we conducted a theoretical (i.e., deductive) thematic analysis for theme mapping. The theoretical thematic analysis described the detail of existing models more appropriately. We constructed our study model based on TTM [13], which is a well-established behavior change theory. At least two authors conducted each analysis step. Therefore, the correspondence ratio of code interpretations was calculated differently from the coauthors’ interpretation, per the first author’s interpretation in steps 3 through 5. The detailed data analysis process is described in Additional file 2.

## Results

We obtained 430 codes (i.e., behavioral or cognitive variables) from the study participants. The mean, minimum, and maximum codes from each participant were *M* = 5.00 (*SD* = 3.55), minimum = 1, and maximum = 18. A total of 21 codes were excluded in steps 1 and 2 (code = 1), step 3 (code = 14), and step 4 (code = 6). None were excluded in step 5 (see Additional file 2). There were 409 total relevant codes. Finally, we identified four themes with 45 subthemes. Additional file 3 shows the results of the original Japanese version. Examples of specific descriptions are reported within “ ” in the manuscript.

Table 2 shows the contents of Theme 1: Health awareness. Health awareness was defined as the recognition of health significance via internal and external resources. Theme 1 consisted of two subthemes, including Awareness of one’s own health and Attention to the health of others. Participants frequently reported “perceive a change in their health” and “Concerned about the illness of close family and friends.”

**Table 2:** Retrieved subtheme, category, and description of theme 1: Health awareness

**Category**	**Examples of description**	**Response**
*Subtheme: Awareness of one’s own health (Total response = 11)*		
Perceived change in health	Perceive a change in their health	4
Anxiety	Worried about their health	3
Pain	Look at the pain in their knee and back	1
Future outlook	Concerning their future	1
Review lifestyle	Review own lifestyle	1
Recognizing the impact on others	Does not want to cause trouble for family and friends	1

*Subtheme: Attention to the health of others (Total response = 11)*		
Attention to the health of family and friends	Concerned about illnesses among close family and friends	5
Interest in health-related conversations	Shows interest in health-related information from friends and acquaintances	4
Health precautions among celebrities	Concerned about information on illness in celebrities and public figures	1
Interest in successful experience	Talk to successful people who have lost weight	1

Table 3 shows the characteristics of Theme 2: Psychological readiness. Psychological readiness was defined as preparative emotional, cognitive, and attitudinal perspectives for engaging in health behavior. Theme 2 was comprised of 13 subthemes, including those that were specific to health behavior (i.e., Interest in physical activity, Consciousness of eating, Reconsideration of smoking, Consciousness of oral care, Attitudinal change toward health checkups, Attention to body weight, Interest in health events, Interest in blood pressure management, and Interest in reducing alcohol consumption) and those that were general (i.e., Attitude toward appearance, Attention to health information, Ownership, and Interest in healthcare devices and applications). Overall, these subthemes reflected emotional arousal and cognitive/attitudinal changes (e.g., “Interested in yoga mats” and “Recognizing that daily lifestyle is linked to future health”).

**Table 3:** Retrieved subtheme, category, and description of theme 2: Psychological readiness

**Specific to health behavior**			**General**		
**Category**	**Example of descriptions**	**Response**	**Category**	**Example of descriptions**	**Response**
*Subtheme: Interest in physical activity (Total response = 22)*			*Subtheme: Attitude toward appearance (Total response = 8)*		
Interest in materials	Interested in yoga mats	7	Attention to abdominal circumference	Concerned about own abdominal circumference (patting the stomach)	3
Introspection of current activity level	Start monitoring number of steps taken	5	Awareness of how they are seen	Concerned about own appearance	2
Interest in physical activity	Start to think about exercising	4	Conscious of clothing and grooming	Start to worry about clothes and hairstyle	2
Interest in facilities	Attach public information about sports gyms	4	Conscious of their body shape	Concerned about their body shape	1
Willingness to go out	Motivated to go out	2			
			*Subtheme: Attention to health information (Total response = 6)*		
*Subtheme: Consciousness of eating (Total response = 17)*			Perception of health information	Attracted to health information on TV, radio, and in the newspaper	4
Awareness of calories	Worry about calories when eating out	7	Intention to collect information	Actively seek to gather information on health promotion	1
Interest in food	Having an interest in foods	6	Interest in health information	Show interest in talking about weight loss	1
Interest in supplements	Curious about supplements	1			
Attention to nutrient composition	Pay attention to nutrient composition	1	*Subtheme: Ownership (Total response = 5)*		
Awareness of salt content	Started to pay more attention to low-sodium products	1	Recognizing the importance of health promotion	Recognizing that daily lifestyle is linked to future health	3
Awareness of carbohydrates	Worry about the amount of rice	1	Internalization of health information	Consider the relationship between media information and their health condition	2
	
*Subtheme: Reconsideration of smoking (Total response = 6)*			*Subtheme: Interest in healthcare devices and applications (Total response = 3)*		
Considering the effects on surroundings	Considering how smoking impacts children	3	Interest in health care devices	Showing interest in materials connected to smartwatches and smartphones	2
Considering effects on the household budget	Wondering about the burden of tobacco on the household budget	2	Knowledge of application	Do not use the application, but understand its function	1
Intention to quit smoking	Thinking about quitting smoking	1			

*Subtheme: Consciousness of oral care (Total response = 3)*					
Halitosis awareness	Worried about bad breath	2			
Interest in oral health care products	Show interest in an electric toothbrush	1			

*Subtheme: Attitudinal change toward health checkups (Total response = 2)*					
Intention to get health checkups	Going to get health checkups	1			
Risk perception	Concern about a similar-aged friend who was retested for cancer screening	1			

*Subtheme: Attention to body weight (Total response = 2)*					
Weight concerns	Worried about body weight	1			
Exploring factors of weight change	Consider factors associated with weight change	1			

*Subtheme: Interest in health events (Total response = 1)*					
Interest in health events	Show interest in health classes	1			

*Subtheme: Interest in blood pressure management (Total response = 1)*					
Interest in sphygmomanometer	Show interest in sphygmomanometer	1			

*Subtheme: Interest to reduce alcohol consumption (Total response = 1)*					
Interest in information on reduced alcohol consumption	Showing interest in information on reduced alcohol consumption	1			

Table 4 shows the characteristics of Theme 3: Gateway behavior. Gateway behavior was defined as precursory behavior that arises prior to engagement in health-related behavior. Theme 3 was similar in structure to theme 2. There were 17 subthemes, including gateway behaviors that were specific to health (Healthy eating, Physical activity, Smoking cessation, Oral care, Health checkups, Health events participation, Weight management, Blood pressure management, Mental health promotion, and Lifestyle improvement) and those that were general (Changing points of discussion, Information gathering, Social participation, Using healthcare devices and applications, Self-analysis, Goal setting, and Stimulus control). Gateway behaviors that were specific to health included those that were not direct health behaviors, but which were still positive behavioral changes, including “looking at food labels while shopping” and “buying a pedometer.” General gateway behaviors were preparative, and without any specific relation to health, including “check body shape in front of the mirror” and “setting a goal to improve health.” As noted, both the health-specific and general gateway behaviors included verbal expressions related to change (e.g., “asking others about health”) and information gathering (e.g., “considering health-related information via the newspaper and TV”).

**Table 4:** Retrieved subtheme, category, and description of theme 3: Gateway behavior

**Specific to health behavior**			**General**		
**Category**	**Example of descriptions**	**Response**	**Category**	**Example of descriptions**	**Response**
*Subtheme: Healthy eating gateway behaviors (Total response = 31)*			*Subtheme: Changing talking content (Total response = 32)*		
Confirming food labels	Looking at food labels while shopping	14	Questioning	Asking others about health	9
Gathering information	Visiting cooking websites	4	Communication	Talk about health with friends and acquaintances	7
Cooking	Stared cooking	4	Self-disclosure	Taking about the experience and thoughts on health-related conversations	5
Dietary recording	Recording the contents of consumed foods	2	Intention to change	Saying “I should change my lifestyle”	2
Change talk	Talk about barriers to improving eating habits	2	Future perspective	Start to talk about the expectations of future health conditions	2
Eating together	Participate in lunch or dinner meetings with aged persons	1	Economical concerns	Talk about large expenses after getting sick	2
Home cultivation	Start growing some vegetables in their home	1	Consultation	Consultation for own physical condition	1
Goal setting	Setting goals about daily calorie intake	1	Expressing a sense of crisis	Saying “I will not make” to their lifestyle	1
Communication	Consults others about food contents	1	Expressing conflict	Comments such as “I cannot do this easily”	1
Tooth brushing	Brushing their teeth soon after dinner to prevent excessive eating	1	Positive talk	Talk becomes positive	1
			Expressing self-efficacy	Talks about possibly they can work for them	1
*Subtheme: Physical activity gateway behavior (Total response = 25)*					
Change talk	Talk about the reflection of physical inactivity	6	*Subtheme: Information gathering (Total response = 16)*		
Material preparation	Buying pedometer	5	Getting from mass media	Seeing health-related information on newspaper and TV	12
Cloths selection	Select a bag that is easy to move, like a backpack or shoulder bag	4	Obtaining books	Renting or purchasing illness-related books	1
Information gathering	Seeing a gymnastic exercise program on TV	3	Read books	Reading related books	1
Seeking companion	Go to a gym with friends or spouse	3	Browsing website	Browsing health-related websites	1
Going out	Increased frequency of going out	2	Using computer	Start to use a computer (including tablet PC) for information gathering	1
Goal setting	Setting a goal about the number of steps	1			
Imagery	Imagining walking route	1	*Subtheme: Social participation (Total response = 3)*		
			Interpersonal communication	An increasing opportunity to meet other people	2
*Subtheme: Smoking cessation gateway behavior (Total response = 6)*			Contact	Contact with others	1
Change talk	Declaring they will quit smoking	2			
Stimulus control	Avoiding shops where smoking is possible	2	*Subtheme: Using healthcare device and application (Total response = 2)*		
Communication	Talking about smoking	1	Purchase healthcare device	Purchases a device that links to smartwatch and phone	1
Change of cigarette type	Switching from cigarettes to an electronic cigarette	1	Installs application on the device	Install healthcare applications on a smartphone	1
	
*Subtheme: Oral care gateway behavior (Total response = 4)*			*Subtheme: Self-analysis (Total response = 2)*		
Purchase oral health products	Purchase electronic toothbrush	2	Confirmation of body shape	Check body shape in front of the mirror	1
Information gathering	Seeking oral health care information	1	Breakdown the self-analysis	Change the answer to the health-related questionnaire as always answered normal to response good or bad	1
Self-observation	Confirm inside of mouth via mirror	1			
			*Subtheme: Goal setting (Total response = 1)*		
*Subtheme: Health checkups gateway behavior (Total response = 3)*			Goal setting	Setting a goal to improve health	1
Change talk	Talking about health checkups with family and friends	2	Action planning	Autonomously think about action to improve their condition	1
Appreciation	Appreciation for health checkups	1			
*Subtheme: Health events participation gateway behavior (Total response = 2)*			*Subtheme: Stimulus control (Total response = 1)*		
			Stimulus control	Putting ideal photograph for them	1
Registration	Registration with health point project	2			

*Subtheme: Weight management gateway behavior (Total response = 1)*					
Purchase scale	Purchase a scale	1			

*Subtheme: Blood pressure measurement gateway behavior (Total response = 1)*					
Purchase sphygmomanometer	Buying a sphygmomanometer	1			

*Subtheme: Mental health promotion gateway behavior (Total response = 1)*					
Information gathering	Seeking stress management techniques	1			

*Subtheme: Lifestyle improvement gateway behaviors (Total response = 1)*					
Daytime activities	Although day-night reversal, waking up during daylight	1			

Finally, Table 5 shows the characteristics of Theme 4: Health behavior related to the contents of traditional healthy lifestyle behaviors. Theme 4 was comprised of 13 subthemes (Eating behavior change, Physical activity, Weight management, Reducing alcohol consumption, Participation in health events, Health checkups, Mental health promotion, Oral care, Smoking reduction, Blood pressure management, Improving life rhythm, Help-seeking behavior, and Trial of health behavior), all of which entailed a low psychological burden and high feasibility.

**Table 5:** Retrieved subtheme, category, and description of theme 4: Health behavior change

**Category**	**Description**	**Response**
*Subtheme: Eating behavior change (Total response = 62)*		
Restriction of carbohydrate and sugar-sweetened food	Choose non-sugar drinks	12
Healthy food choice	Consciously consume foods that look healthy	8
Vegetable intake	Eating veritable every meal	7
Restricting salt content	Making a lightness of taste	6
Having regular meals	Eating all meals regularly	5
Considering order to eat	Eating vegetables first	5
Restricting between-meal eating	Reducing between-meal eating	5
Balance	Become more aware of nutrient balance.	4
Calorie restriction	Change snacks to go with alcohol toward healthy products (green beans, tofu, and vegetables)	4
Ingenuity in eating	Eating moderately	4
Limiting fat	Reduce fat intake	1
Increasing total meal intake	People who could not intake enough nutrients begin to eat well	1

*Subtheme: Physical activity (Total response = 46)*		
Walking	Walking ten minutes per day	11
Daily walking	Park a car far from the entrance of the market for walking	10
Using stairs	Using stairs instead of the elevator	10
Go to sport facilities	Start to go to the sports gym	4
Stretching	Try to do stretching exercises introduced on a TV program	3
Lifestyle physical activity	Going shopping every day	2
Brisk physical activity	Physically active during in-between time	2
Running	Go for a run	1
Bicycle use	Ride a bicycle	1
Yoga	Start to do yoga	1

*Subtheme: Weight management (Total response = 14)*		
Body weight management	Measuring body weight every day	14

*Subtheme: Reducing alcohol consumption (Total response = 11)*		
Reducing alcohol consumption	Select smaller can sizes	5
Setting a non-alcohol day	Establish a rest day of drinking	4
Selected purchase of ingredients	Select sugar-free alcohol products	1
Stop holding a reserve	Stop a stock of alcohol beverages	1

*Subtheme: Participation in health events (Total response = 11)*		
Participate in health events	Participate in health events	11

*Subtheme: health checkups (Total response = 9)*		
Getting health checkups	Getting health checkups	9

*Subtheme: Mental health promotion (Total response = 8)*		
Taking a rest	Take a holiday for refreshment	3
Communication	Consultations to avoid frustration and compliment	2
Hobbies	Engage in hobbies for stress reduction	2
Sleeping	Keep sleeping time	1

*Subtheme: Oral care (Total response = 5)*		
Gettering a dental examination	Getting a dental examination	2
Practicing oral health care	Use dental floss	2
Use of chewing gum	Chewing gum with xylitol	1

*Subtheme: Smoking reduction (Total response = 4)*		
Reducing cigarette use	Reduce the number of cigarettes used per day	4

*Subtheme: Blood pressure measurement (Total response = 3)*		
Measuring blood pressure	Measuring blood pressure	3

*Subtheme: Improving life rhythm (Total response = 2)*		
Regularization of life	Maintain a regular rhythm of life	1
Quit staying up late	Stop staying up late	1

*Subtheme: Help-seeking behavior (Total response = 2)*		
Communicate with health professionals	Meets with health professionals	2

*Subtheme: Trial of health behavior (Total response = 1)*		
Trial of health behavior	Trying to engage in any health behaviors	1

**Fig. 1:**
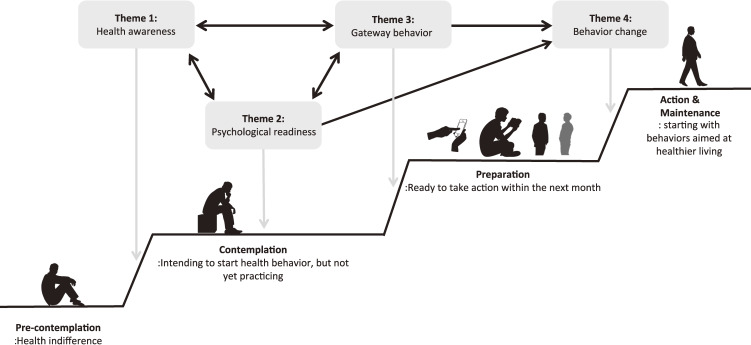
Theme map of the stage of changes process in individuals who are uninterested in health This theme map is a proposed model for lower-stage behavior changes in individuals who are uninterested in health.

As shown in Fig. 1, the thematic map of the model for the behavior change process in the initial stages among the UPH population was constructed according to the present study results and TTM model. Although we could not establish a causal relationship, health awareness (Theme 1) may arise first during the processual stage moving from pre-contemplation to contemplation. Increasing awareness of health conditions for themselves and others may be linked to increased psychological readiness for health care (Theme 2) and gateway behavior change (Theme 3). These themes interact during the pre-contemplation to preparation stages. In addition, psychological readiness (Theme 2) and gateway behavior (Theme 3) were situated in the contemplation stage. The contemplation and preparation stage refers to the intention to start but not yet practicing health behavior. However, it may already act as a preparative behavior due to the slight interest in lifestyle changes. These themes may work as preceding factors for small health behavior changes in the preparation to action stage (Theme 4).

## Discussion

This study explored the behavior change process in UPH based on professional knowledge and expertise. Our thematic analysis identified four main themes, including health awareness, psychological readiness, gateway behavior, and health behavior, with 45 subthemes. We then constructed a thematic map based on TTM. Thus, we created a model for the behavior change process in the lower stage population.

The most significant finding of our qualitative analysis was that health awareness may be an essential factor in health behavior changes among UPH. Although traditional behavioral change intervention focuses on increasing the motivation, self-efficacy, and behavioral intention of the general population [16, 25], promoting health awareness may be an essential first step for UPM. The health behavior change continuum model begins with the awareness phase, including the internalization of health information [26]. A previous study found evidence of relationships between negative/positive self-awareness and health behaviors/outcomes [27]. According to the health belief model [28], perceived susceptibility, seriousness, and threat to adverse health outcomes predict preventive health action. Another study also reported that self-evaluations of body appearance were associated with proper nutrition behaviors and positive attitudes toward health behavior [29]. Awareness of one’s own and health and that of close others also has the potential to be highly relevant information. The perceived health condition of close relations such as family and friends may work as a significant and substantial resource for cognition and attitude changes [30].

Of particular note, we identified that both Theme 2: Psychological readiness and Theme 3: Gateway behavior were precursory cognitive and behavioral concepts that preceded the practice of health-related behaviors. These themes may therefore be regarded as gateways to health behavior changes. Schwandt et al. [23] developed a gateway model focused on slight precursory psychological and behavioral changes to health behavior, therein showing that small emotional, cognitive, and behavioral changes occurred during the gateway moment (e.g., significant experiences in developmental stages or life events); here, a gateway behavior that shows preliminary interest in health-oriented action emerges. However, empirical research has not considered specific cognitive changes and gateway behaviors. As such, this study identified specific cognitive changes and the details of gateway behaviors. Specifically, many participants frequently reported changes in points of discussion and the active search for relevant information. This finding implies the significance of talk that is related to change, as reflected by the well-established motivational interviewing framework [31]. This type of talk implies the motivation for change, desire for health, ability, reasons, needs, and commitment to changing one’s health behavior [32]. Likewise, we found that access to health information may also be a significant factor that is related to change. Several studies have reported that health exposure in the media increases health awareness [33]. In addition, Lambert and Loiselle [34] found that seeking health information had positive effects on emotional, cognitive, and behavioral changes while increasing QoL.

Based on the TTM, this study constructed a model for the process of behavior change in lower stages among the UPH population. Previous meta-analyses have shown that the advanced effect of the stage-matched approach to encourage health behavior was unclear when compared to the non-matched intervention [35]. However, few studies have considered differences in behavior change strategies according to the stage. The constructed model indicated no equivalence of the behavior change process of pre-contemplation (i.e., UPH) to the contemplation stage and contemplation to the upper stage. Changing pre-contemplation to contemplation requires increasing awareness of our own and essential others’ health. On the other hand, contemplation changing to the upper stage needs to reinforce traditional psychological and behavioral variables such as motivation, self-efficacy, behavioral intention, and small lifestyle changes.

Behavior change programs are rooted in evidence-based techniques. In particular, setting goals and establishing action plans are critical first steps to encouraging behavior change [36]. Although other studies have emphasized it is difficult to create action plans in the UPH context [37]. These inconsistencies suggest that traditional health education strategies and models are not sufficient for inducing or explaining the behavior change process in UPH. Setting goals and creating action plans may be premature in UPH. We speculate that individuals in the pre-contemplation stage require a specialized intervention strategy. In this regard, a comprehensive meta-analysis showed that both self-monitoring (i.e., the experience of facing oneself) and perceiving health issues as a personal matter primarily contributed to behavior change [38]. As part of an essential intervention strategy, it may therefore be critical to promote self-monitoring in UPH.

This study had several limitations that require further research and practical consideration. First, the thematic analysis and model construction were based on professional health experience. There was also the existing risk of an indirectness bias. The uncertain quality of the evidence elicited from expert opinions was considered [39]. Although the qualitative data were based on professional experience and sufficiently reflected the characteristics of UPH, there may be several gaps between actual psychological and behavioral statuses in the actual target audience and the present study results. Second, the participants of this study were female public health nurses and early career professionals. Thus, a sampling bias may affect the results of the research. Third, the survey was indirectly assessed for UPH. Therefore, personal background information of UPH (i.e., residential area, family backgrounds, and other demographic characteristics) was not considered. Fourth, the study questionnaire simply described the definition of UPH as “People who are not interested in health.” Therefore, study participants might interpret UPH in slightly different ways (i.e., risk of bias according to the reactivity of information) [40]. Fifth, this study implemented qualitative research methods to extract psychological and behavioral factors during the behavior change process. Thus, the causal relationship is still unknown. This highlights the need for longitudinal quantitative studies to identify any relationships between factors. Fifth, this study did not consider personal background factors (i.e., physical/psychological states and socioeconomic status). These factors should be considered in future research, as personal backgrounds may significantly influence health promotion programs for UPH [41, 42].

## Conclusion

Behavior changes in UPH are among the most significant concerns in the healthcare setting. However, it is difficult to encourage health behaviors in this population, meaning they are often left behind in the context of healthcare policies and systems, especially when health professionals resign their efforts. This study clarified the specific cognitive-behavioral characteristics of UPH and constructed a model for their behavior change process, as these issues have received very little attention in the literature. Our findings constitute fundamental knowledge that health professionals can use to encourage UPH to engage in healthy lifestyles. Future studies should aim to increase the reliability and validity of our study variables and model via quantitative and longitudinal approaches. Moreover, sophisticated statistical analyses may offer causal insights into the dose-response relationships between the primary psychological and behavioral variables.

## Abbreviations

UPH: uninterested people toward health
QoL: quality of life
TTM: Transtheoretical Model
STD: self-determination theory
WHO: World Health Organization
MHLW: Ministry of Health, Labour and Welfare
SRQR: standards for reporting qualitative research

## Supplementary information

**Additional file 1:** Standards for Reporting Qualitative Research (SRQR)*.

**Additional file 2:** The detailed thematic analysis process.

**Additional file 3:** The results of the original Japanese version of tables.

## Declarations

### Availability of data and material

The data collected in this study are not publicly available due to ethical concerns. However, they may be made available upon reasonable request to the corresponding author.
