# Supplementary material for: The process of behavioral change in individuals who are uninterested in health: a qualitative study based on professional health knowledge
Source: Environ Health Prev Med. 2022 Jul 28;27:32. doi: 10.1265/ehpm.22-00072 (PMC9357552; doi:10.1265/ehpm.22-00072)
Supplement: Supplementary file 2 — Additional file 2: The detailed thematic analysis process. [file ehpm-27-032-s002.docx]

**Additional file 2. The detailed thematic analysis process**

**Terminological definitions in the current thematic analysis**

This study defined the following classifications: Theme: primary component of the theoretical model, subtheme: cognitive or behavioral unit consolidated category, category: integrated data from codes via similar meaning, and code: short sentences retrieved from descriptive data referring to specific cognitive or behavioral phenomena.

**Overview of the data analysis and authors’ contributions**

Five researchers engaged in the data analysis to enhance trustworthiness and interpretability. The first author (T.S.) is an experienced Ph.D. level health psychologist who has published qualitative research. T.S. entered the descriptive data into the word processor and synthesized codes from the entire law dataset (steps 1 and 2). T.S. and the second author (H.O.), who is a medical doctor specializing in social medicine, processed the first step by coding and identifying the theme (step 3). T.S. and a Ph.D. level epidemiologist (T.Y.) reviewed the synthesized primary and subtheme (step 4). After modifying the classifications, provisional theme, and preliminary model, T.S. (professor of public health; M.S.) and health psychology (K.T.) reviewed the overall analysis results and reconsidered the thematic map (step 5). This study conducted a theoretical thematic analysis, which is an existing method of theory-based mapping [23]. The TTM model [13] was reasonable for interpreting retrieved themes. After supervising and modifying the data analysis results, all authors confirmed the theme and thematic map definitions. T.S. reported the overall results in the research article (step 6). The correspondence ratio of code interpretation in steps 3, 4, and 5 were 95.8% (inconsistence code = 18), 98.3% (inconsistence code = 7), and 99.0 % (inconsistence code = 5), respectively.

**Ⅰ．Steps 1 and 2: Coding and potential subtheme identification, by T.S. (August 10, 2021)**

This study used inductive methods of thematic analysis. A total of 430 codes were retrieved from the descriptive law data. One code did not match the present research aim (Thinking and caring about their children). Overall, the first analysis step thus resulted in 429 codes.

The first author (T.S.) identified 19 potential subthemes, as follows: eating behavior (code = 110), physical activity (code = 95), communication (code = 30), health checkups (code = 27), information collection (code = 24), self-health awareness (code = 20), body shape/weight management (code = 20), smoking cessation (code =16), participate in health related events (code = 14), oral hygiene (code = 12), reduced alcohol consumption (code =12), other awareness of health (code = 11), attention to own appearance (code = 10), active rest and mental health promotion (code = 9), manage blood pressure (code =5), interest in and use of health encouraging mobile applications (code = 5), social participation (n = 4), improve regularity of life (code = 3), trial of health behavior (code = 1), and reconsidering insurance (code = 1).

**Ⅱ．Step 3: Reconsidering codes and subthemes, by T.S. and H.O. (August 30, 2021–September 27, 2021)**

The second author (H.O.), who is a medical doctor who specializes in social medicine, reviewed the codes and potential subthemes (i.e., step 1 results).

***Code consideration***

A total of 18 codes had inconsistent interpretations (correspondence ratio = 95.8 %).

(1) Talk about Pokémon GO (a mobile application that encourages physical activity)

Reason: It is not directly connected with health.

(2) Buying salad in the convenience store

Reason: The context of the situation was unclear.

(3) Concerned with own clothing and hairstyle

Reason: It is not linked with health.

(4) Reconsidering voluntary insurance

Reason: It is not sufficient in the general sense, as disadvantaged populations may not be able to obtain voluntary insurance. In addition, voluntary insurance may be reconsidered during certain life events.

(5) Health checkup related answers (13 codes)

Reason: A total of 13 codes implied positive changes after receiving health checkups (e.g., concerned about the results of health checkups, imagined an illness upon seeing health checkup results).

After considerable discussions between T.S. and H.O., the step 2 analysis eliminated 14 codes: (4) Reconsidering voluntary insurance and (5) Health checkup related answers (13 codes). As such, there were 415 analysis codes. In addition, T.S. reconsidered eight codes for interpretation and modified assignments.

***Subtheme consideration***

Inconsistent interpretation provisional subtheme (subtheme = 5)

(1) Body shape/weight management

Reason: Body shape and weight management may constitute a different category of concern, as each may include appearance anxiety.

(2) Appearance

Reason: Appearance may be connected to the body shape/weight management subtheme.

(3) Reconsidering voluntary insurance

Reason: Same as the reason given in the code consideration process.

(4) Communication

Reason: A broad meaning was included in the code. Here, change in discussions and social participation should be separated, thus contributing to a more nuanced understanding.

After discussions between T.S. and H.O., voluntary insurance was excluded.

The potential subthemes were reconstructed as follows: “Body shape/weight management” was reclassified as concern about body shape and weight management behavior. In addition, concern about body shape-related codes were integrated with “appearance.” The potential subtheme “Reconsidering voluntary insurance” was eliminated from the data analysis. In addition, “Communication” was divided into two new subthemes. “Speech” is related to positive statements concerning a healthy lifestyle and discussions about conflicts pertaining to health behavior change (i.e., change talk). The “Social participation” subtheme refers to interest and trial related to social participation and communicating with other persons. According to the step 3 analysis results, T.S. preliminary defined the three following themes: Theme 1: Attitude change, Theme 2: Health behavior-specific behavior change, and Theme 3: general behavior change.

**Ⅲ. Step 4: Reviewing codes, subthemes, and the provisional model (October 14, 2021)**

A Ph.D. epidemiologist (T.Y.) reviewed the results of the previous steps. T.Y. made several modifications to the codes, subthemes, and models.

**Theme consideration**

The step 4 results showed different theme interpretations from the step 3 results. It was reasonable to distinguish between health behavior and health gateway behavior as well as cognition that seemed to change health behavior. For instance, paying attention to calories, interest in physical activity facilities, imagining the practice of healthy behavior, and goal setting may occur before health behaviors change. Therefore, the themes were reconstructed into the four following domains: Theme 1: Attitude change, Theme 2: Health behavior-specific gateway cognition and behavior, Theme 3: General gateway cognition and behavior, and Theme 4: Health behavior change.

**Code consideration**

T.Y. noted inconsistent explanations in seven codes.

(1) Recording physical activity log (three codes)

Reason: Recording behavior may express after behavior change. This would contribute to habit formation. However, recording is not a gateway. Therefore, three codes related to the physical activity log were excluded in step 4.

(2) Measuring physical condition

Reason: The measurement method and type is ambiguous. Therefore, this code was excluded.

(3) Recording body weight (two codes)

Reason: The reason is the same as that given above for recording physical activity log; recording body weight may occur after the behavior change phase. Two codes were eliminated.

(4) Day-night reversal, but efforts to wake during the day

Reason: This behavior implies an attempt to improve life rhythm. However, it may be insufficient to call this a health behavior. Therefore, “Day-night reversal, but effort to wake during day” was moved to gateway behavior.

(6) Setting goals for improved health

Reason: The practice of goal setting has the potential to work as a significant gateway for independent behavior. Therefore, “Goal setting to improve health” was set as an independent category in ownership as a subtheme in the general gateway dimension.

(7) Meets health professionals

Reason: This code was defined as a category related to communication with professionals in the subtheme of social participation and speech. However, meeting health professionals is associated with heavy psychological burdens. Regarding help-seeking behavior that stands subtheme in theme 4: Health behavior change seems better for reasonable interpretation.

A total of 409 analysis codes remained after eliminating six.

**Subtheme consideration**

T.Y. agreed to perform the step 3 subtheme classifications. Only “Goal setting” was added as a subtheme in the general gateway dimension.

**Ⅳ．Step 5: Reconsidering the overall theme codes, themes, and thematic map (October 20, 2021)**

**Theme consideration**

M.S. and K.T. asked to reconsider the overall theme definitions according to the cognitive­–behavioral relationship. M.S. indicated that cognitive and behavioral variables assigned to the independent themes may allow for a better understanding of the phenomenon of behavior change among individuals in the health indifference population. In addition, the name of Theme 1, “self-awareness,” was regarded to reflect participants’ responses. After reconsidering the overall themes, the definitions were reconceptualized as theme 1: Health awareness, theme 2: Increased psychological readiness to change, theme 3: Gateway behavior change, and theme 4: Health behavior change.

**Code consideration**

Six codes were modified in this step. Five codes in the subtheme of ownership had inconsistent interpretations. These codes reflected the intention to change behavior and indicated cognitive activity. Therefore, five codes were moved into Theme 2: Psychological readiness to change and Theme 3: Gateway behavior change. In addition, the “application” code in the subtheme of getting health checkups in Theme 4 pertained to gateway behavior. The “appreciation” code was moved to Theme 3.

**Subtheme consideration**

Regarding theme consideration, three subthemes were reconsidered and the names of 14 subthemes were changed. The three modified subthemes are as follows:

(1) Subtheme: Ownership in Theme 2

Ownership was defined as a subtheme of Theme 1: Attitude in the step 4 analysis. However, the two codes of “perceived importance of health promotion” and “internalization of health information” were regarded as psychological readiness to change. These two codes were moved to Theme 2 and constructed as new subthemes.

(2) Subtheme: Confirmation of body shape and stimulus control

Confirmation of body shape and stimulus control were regarded as gateway behaviors or appearance concerns in step 4. However, these codes were regarded as different types of behavior. Therefore, confirmation of body shape and stimulus control were redefined as independent subthemes.

**Thematic map construction**

After reconsidering the themes, codes, and subthemes, the first author constructed a thematic map according to the Transtheoretical Model. The other authors checked the constructed model and made a few revisions to simplify the theme names, as follows: Theme 1: Health awareness, Theme 2: Psychological readiness, Theme 3: Gateway behavior, and Theme 4: Behavior change. In addition, the first author defined each theme and described them in the manuscript.
