# Supplementary material for: The process of behavioral change in individuals who are uninterested in health: a qualitative study based on professional health knowledge
Source: Environ Health Prev Med. 2022 Jul 28;27:32. doi: 10.1265/ehpm.22-00072 (PMC9357552; doi:10.1265/ehpm.22-00072)
Supplement: Supplementary file 3 — Additional file 3: The results of the original Japanese version of tables. [file ehpm-27-032-s003.docx]

**Additional File 3. The results of the original Japanese version of tables**

Table 2 健康意識（テーマ1）

| カテゴリー | 回答の例 | Response |
| --- | --- | --- |
| *サブテーマ：自己の健康に対する意識(Total response = 11)* | |  |
| 健康面での変化の認知 | 健康面での変化を実感する | 4 |
| 不安 | 体調に不安を感じる | 3 |
| 痛み | 膝や腰の痛みに目が向く | 1 |
| 将来展望 | 将来のことを考える | 1 |
| 生活習慣の見直し | 自分自身の生活習慣を見直す | 1 |
| 他者への影響の認知 | 家族や友人に迷惑をかけないようにと思う | 1 |
|  |  |  |
| *サブテーマ：他者の健康に対する注意(Total response = 11)* | |  |
| 家族・友人の健康に対する注意 | 身近な人の病気（家族・友人）を気にする | 5 |
| 健康に関する話題への興味 | 友人・知人からの健康に関する情報に関心を示す | 4 |
| 著名人の健康に対する注意 | 芸能人・有名人の病気の報道を気にする | 1 |
| 成功体験への興味 | 「痩せた」など成功者に話を聞く | 1 |

Table 3　心理的レディネス（テーマ2）

| 健康行動特異的項目 | | |  | 一般性項目 | | |
| --- | --- | --- | --- | --- | --- | --- |
| カテゴリー | 回答の例 | Response |  | カテゴリー | 回答の例 | Response |
| *サブテーマ：身体活動への興味(Total response = 22)* | |  |  | *サブテーマ：外見に対する意識(Total response = 8)* | |  |
| 用具への関心 | ヨガマットに興味を持つ | 7 |  | 腹囲に対する意識 | 腹囲を気にする（お腹をなでる） | 3 |
| 現在の活動量の内省 | 歩数を気にしだす | 5 |  | 見られ方への意識 | 見た目を気にする | 2 |
| 活動への関心 | 運動しようと考え始める | 4 |  | 服装や身だしなみへの意識 | 服装や髪型を気にしだす | 2 |
| 施設への興味 | スポーツジムの広報を見る | 4 |  | 体型への意識 | 体型を気にする | 1 |
| 外出への意欲 | 外出する気が湧く | 2 |  |  |  |  |
|  |  |  |  | *サブテーマ：健康情報への注意 (Total response = 6)* | |  |
| *サブテーマ：食への意識(Total response = 17)* | |  |  | 健康情報の知覚 | テレビ・ラジオ・新聞の健康情報を目に止める | 4 |
| カロリーへの意識 | 外食時にカロリーを気にする | 7 |  | 情報収集の意図 | 積極的に健康づくりに関する情報を集めようとする | 1 |
| 食事への興味・関心 | 食べ物に興味を示す | 6 |  | 健康情報への興味 | 減量に関する話に興味を示す | 1 |
| サプリメントへの興味 | サプリメントが気になる | 1 |  |  |  |  |
| 栄養成分への意識 | 栄養成分を気にするようになる | 1 |  | サブテーマ：当事者意識（Total response = 5) | |  |
| 塩分への意識 | 減塩食品をきにするようになった | 1 |  | 健康づくりの重要性の認知 | 「日々の生活習慣の積み重ねが将来の健康につながる」と認識する | 3 |
| 炭水化物への意識 | ご飯の量を気にする | 1 |  | 健康情報の内在化 | メディアの健康情報と自分の健康状態を関連して考える | 2 |
|  |  |  |  |  |  |  |
| *サブテーマ：喫煙行動の再考(Total response = 6)* | |  |  | *サブテーマ：支援機器・アプリへの興味（Total response = 3)* | |  |
| 周囲への影響の考慮 | 喫煙の子どもへの影響を考える | 3 |  | 支援機器の興味 | スマートウォッチ・スマホとリンクする機器に興味を示す | 2 |
| 家計への影響考慮 | タバコの家計への負担について考える | 2 |  | アプリに関する知識 | アプリを使ってはいないが機能を知っている | 1 |
| 禁煙意図 | 禁煙を考える | 1 |  |  |  |  |
|  |  |  |  |  |  |  |
| *サブテーマ：口腔ケアへの意識(Total response = 3)* | |  |  |  |  |  |
| 口臭の意識 | 口臭を気にする | 2 |  |  |  |  |
| 口腔ケア用品への興味 | 電動歯ブラシに興味を示す | 1 |  |  |  |  |
|  |  |  |  |  |  |  |
| *サブテーマ：健康診査受診への態度変容（Total response = 2)* | |  |  |  |  |  |
| 受診意図 | 検診を受診しようと思う | 1 |  |  |  |  |
| リスク認知 | がん検診で再検査となった近い年齢の友人を気にする | 1 |  |  |  |  |
|  |  |  |  |  |  |  |
| *サブテーマ：体重への意識(Total response = 2)* | |  |  |  |  |  |
| 体重の心配 | 体重を気にする | 1 |  |  |  |  |
| 体重変化の要因探索 | 自身の体重変化について要因を考える | 1 |  |  |  |  |
|  |  |  |  |  |  |  |
| *サブテーマ：健康イベントへの興味(Total response = 1)* | |  |  |  |  |  |
| 健康イベントへの興味 | 健康講座に興味を示す | 1 |  |  |  |  |
|  |  |  |  |  |  |  |
| *サブテーマ：血圧管理への興味(Total response = 1)* | |  |  |  |  |  |
| 血圧計への興味 | 血圧計に興味を示す | 1 |  |  |  |  |
|  |  |  |  |  |  |  |
| *サブテーマ：節酒への興味(Total response = 1)* | |  |  |  |  |  |
| 節酒情報への興味 | 節酒の情報に興味を示す | 1 |  |  |  |  |

Table 4　ゲートウェイ行動（テーマ3）

| 健康行動特異的項目 | | | 一般性項目 | | |
| --- | --- | --- | --- | --- | --- |
| カテゴリー | 回答の例 | Response | カテゴリー | 回答の例 | Response |
| *サブテーマ：食ゲートウェイ行動 (Total response = 31)* | | | *サブテーマ:発話の変化(Total response = 32)* | | |
| 食品表示確認 | 買い物の時に成分表を見るようになった | 14 | 質問 | 健康に関して他者に質問する | 9 |
| 情報収集 | 料理のサイトを見る | 4 | 周囲とのコミュニケーション | 友人知人に健康に関する話をする | 7 |
| 調理行動 | 料理を始めた | 4 | 自己開示 | 健康に対する話題提供に対し，自分のことを話し始める | 5 |
| 食事記録 | 食事の内容を記録する | 2 | 変化への意志 | 「やっぱり生活を変えていかないとね」と言葉に出す | 2 |
| チェンジトーク | 食事改善に対しての阻害要因を口にする | 2 | 将来の展望 | 将来の健康状態について話し始める | 2 |
| 共食 | 老人会や独居老人の食事会に参加する | 1 | 経済的な心配 | 病気になるとお金がかかると話す | 2 |
| 家庭栽培 | 自宅で野菜作りを始める | 1 | 相談 | 自身の体調について相談する | 1 |
| 目標設定 | 1日の摂取カロリーに対して目標を設定する | 1 | 危機感の表出 | 生活習慣に対して「何かしないとまずい」という発言がある | 1 |
| コミュニケーション | 食事の中身について相談する | 1 | 葛藤表出 | 「なかなかできない」と弱音や愚痴を言う | 1 |
| 歯磨き | 食べ過ぎ防止のために早めに歯を磨く | 1 | 前向きな発話 | 話す内容が前向きになった | 1 |
|  |  |  | 自己効力的発話 | 自分でもできそうなことについて話しをする | 1 |
| *サブテーマ：身体活動ゲートウェイ行動 (Total response = 25)* | | |  |  |  |
| チェンジトーク | 不活動を反省する発言をする | 6 | *サブテーマ：情報収集 (Total response = 16)* | | |
| 用品準備 | 万歩計を購入する | 5 | マスメディアからの入手 | 新聞やテレビで健康関連情報を見る | 11 |
| 服装選択 | リュックや肩掛けなどの動きやすいカバンを選ぶ | 4 | 書籍の入手 | 病気に関する図書の貸出・購入 | 1 |
| 情報収集 | テレビの体操番組を見ている | 3 | 書籍の閲読 | 関連する本を読む | 1 |
| 仲間探し | 友人や配偶者と一緒にジムに行く | 3 | ラジオの視聴 | 健康関連のラジオを聴く | 1 |
| 外出 | 外出の機会が増える | 2 | ウェブサイトの閲覧 | 健康関連のウェブサイトを見る | 1 |
| 目標設定 | 目標歩数を設定する | 1 | PCの使用 | 情報収集のためにPC（タブレット）を使い始める | 1 |
| イメージ | 散歩やウォーキングのルートをイメージする | 1 |  |  |  |
|  |  |  | *サブテーマ：社会参加(Total response = 3)* | | |
| *サブテーマ：禁煙ゲートウェイ行動(Total response = 6)* | | | 対人コミュニケーション | 人と会う機会が増える | 2 |
| チェンジトーク | 禁煙を宣言する | 2 | 連絡 | 連絡を取る | 1 |
| 刺激統制 | 喫煙可の店舗を避ける | 2 |  |  |  |
| コミュニケーション | 喫煙者がたばこについて話す | 1 | *サブテーマ：支援機器・アプリの利用(Total response = 2)* | | |
| 種類の変更 | タバコから電子タバコにかえる | 1 | 支援機器の購入 | スマートウォッチ・スマホとリンクする機器を購入する | 1 |
|  |  |  | アプリのインストール | スマートフォンに健康アプリを入れる | 1 |
| *サブテーマ：口腔ケアゲートウェイ行動(Total response = 4)* | | |  |  |  |
| 口腔ケア用品の購入 | 電動歯ブラシを購入する | 2 | *サブテーマ：自己分析(Total response = 2)* | | |
| 情報収集 | 口腔ケア情報を調べる | 1 | 体型確認 | 鏡で体型を確認する | 1 |
| 自己観察 | 鏡で口の中を確認する | 1 | 自己分析の細分化 | 健康状態に関する質問票に普通と書いていたのが，良い，悪いと書くようになる | 1 |
|  |  |  |  |  |  |
| *サブテーマ：健康診査受診ゲートウェイ行動 （Total response = 3)* | | | *サブテーマ：目標設定(Total response = 1)* | | |
| チェンジトーク | 家族や友人に健康診査の話をする | 2 | 目標設定 | 健康改善の目標を立てる | 1 |
| 申し込み | 検診を申し込む | 1 | 行動計画 | 体調を整えるために自発的に行えることを考える | 1 |
|  |  |  |  |  |  |
| *サブテーマ：健康事業参加ゲートウェイ行動 (Total response = 2)* | | | *サブテーマ：刺激統制（Total response = 1）* | | |
| 参加登録 | 健康ポイント事業に登録する | 2 | 刺激統制 | 理想の写真を貼る | 1 |
|  |  |  |  |  |  |
| *サブテーマ：体重管理ゲートウェイ行動 (Total response = 1)* | | |  |  |  |
| 体重計の購入 | 体重計を購入する | 1 |  |  |  |
|  |  |  |  |  |  |
| *サブテーマ：血圧測定ゲートウェイ行動(Total response = 1)* | | |  |  |  |
| 血圧計の購入 | 血圧計を購入する | 1 |  |  |  |
|  |  |  |  |  |  |
| *サブテーマ：メンタルヘルスプロモーションゲートウェイ行動 (Total response = 1)* | | |  |  |  |
| 情報収集 | ストレス発散法を見つける | 1 |  |  |  |
|  |  |  |  |  |  |
| *サブテーマ：生活リズム改善ゲートウェイ行動（Total response = 1)* | | |  |  |  |
| 日中の活動 | 昼夜逆転の生活だが日中起きている | 1 |  |  |  |

Table 5　健康行動変容（テーマ4）

| カテゴリー | 回答の例 | Response |
| --- | --- | --- |
| *サブテーマ：食行動 (Total response = 62)* |  |  |
| 炭水化物・糖質制限 | 飲み物は無糖を選ぶ | 12 |
| 健康的な食の選択 | 健康に良さそうな食品を意識的に摂る | 8 |
| 野菜摂取 | 毎食野菜を摂る | 7 |
| 塩分の制限 | 薄めの味付けにする | 6 |
| 食事の規則性 | 決まった時間に食事をする | 5 |
| 食事の順序 | 野菜から食べるようにする | 5 |
| 間食の制限 | 間食を減らす | 5 |
| バランス | 栄養バランスに気をつけるようになる | 4 |
| カロリー制限 | お酒のつまみをヘルシーなもの（枝豆・豆腐・野菜）にする | 4 |
| 食べ方の工夫 | 腹八分目にする | 4 |
| 脂質の制限 | 脂質を控える | 1 |
| 食事量の増加 | 食事を摂らなかった人が食事を摂るようになる | 1 |
|  |  |  |
| *サブテーマ：身体活動 (Total response = 46)* |  |  |
| ウォーキング | 1日10分のウォーキング | 11 |
| 日常生活歩行 | 駐車場で入口から遠いところに置くようにする | 10 |
| 階段利用 | EVを使わず階段を使う | 10 |
| スポーツ施設の利用 | スポーツジムに通い始める | 4 |
| ストレッチ | 健康番組で紹介している体操を試す | 3 |
| 生活活動 | 毎日買い物に行く | 2 |
| 細切れ運動 | 隙間時間で身体を動かすようになる | 2 |
| ランニング | ランニングをする | 1 |
| 自転車利用 | 自転車に乗る | 1 |
| ヨガ | ヨガを始める | 1 |
|  |  |  |
| *サブテーマ：体重管理 (Total response = 14)* |  |  |
| 体重測定 | 毎日体重を測る | 14 |
|  |  |  |
| *サブテーマ：節酒 (Total response = 11)* |  |  |
| 減酒 | 飲酒する缶のサイズを小さいものにする | 5 |
| 休肝日の設定 | 休肝日を設ける | 4 |
| 成分を考慮した選択購入 | 糖質オフのアルコール類を選ぶ | 1 |
| 買い置きの停止 | お酒の買い置きをやめる | 1 |
|  |  |  |
| *サブテーマ：健康イベントへの参加 (Total response = 11)* | |  |
| 健康イベントへの参加 | 健康イベントに参加する | 11 |
|  |  |  |
| *サブテーマ：健康診査受診 （Total response = 9)* |  |  |
| 受診 | 健診を受診する | 9 |
|  |  |  |
| *サブテーマ：休養・心の健康 (Total response = 8)* |  |  |
| 休養 | リフレッシュに休暇を取る | 3 |
| コミュニケーション | 愚痴や不満をためず相談する | 2 |
| 趣味活動 | ストレス解消のために趣味活動を実践する | 2 |
| 睡眠 | 睡眠時間を確保する | 1 |
|  |  |  |
| *サブテーマ：口腔ケア(Total response = 5)* |  |  |
| 受診 | 歯科検診を受診する | 2 |
| 口腔ケアの実施 | 糸ようじを使う | 2 |
| ガムの利用 | キシリトールガムを食べる | 1 |
|  |  |  |
| *サブテーマ：禁煙(Total response = 4)* |  |  |
| 減煙 | 1日の喫煙本数を減らす | 4 |
|  |  |  |
| *サブテーマ：血圧測定(Total response = 3)* |  |  |
| 血圧の測定 | 血圧を測定する | 3 |
|  |  |  |
| *サブテーマ：生活リズム改善（Total response = 2)* |  |  |
| 生活の規則化 | 規則正しい生活リズムを送る | 1 |
| 夜更かしの停止 | 夜更かしをやめる | 1 |
|  |  |  |
| *サブテーマ：援助要請行動(Total resepose = 2)* |  |  |
| 専門家とのコミュニケーション | 専門家に会いに来る | 2 |
|  |  |  |
| *サブテーマ：行動の試行 (Total response = 1)* |  |  |
| 行動の試行 | 何か一つでも健康行動を試してみる | 1 |
